# Experimental Study on the Comparison between Network Microstructure Titanium Matrix Composites and Ti6Al4V on EDM Milling

**DOI:** 10.3390/ma17102282

**Published:** 2024-05-11

**Authors:** Leheng Zhang, Yizhou Hu, Sirui Gong, Zhenlong Wang

**Affiliations:** 1 School of Mechatronics Engineering, Harbin Institute of Technology, Harbin 150001, China; 22s008119@stu.hit.edu.cn (L.Z.); 21b908123@stu.hit.edu.cn (Y.H.); 19b908132@stu.hit.edu.cn (S.G.); 2 Key Laboratory of Micro-Systems and Micro-Structures Manufacturing of Ministry of Education, Harbin Institute of Technology, Harbin 150001, China

**Keywords:** EDM milling, NMTMCs, Ti6Al4V, surface roughness, recast layer thickness, recast layer thickness range, surface microcrack density

## Abstract

Network microstructure titanium matrix composites (NMTMCs), featuring Ti6Al4V as the matrix and network-distributed TiB whiskers (TiBw) as reinforcement, exhibit remarkable potential for diverse applications due to their superior physical properties. Due to the difficulty in machining titanium matrix composites, electrical discharge machining (EDM) stands as one of the preferred machining techniques for NMTMCs. Nevertheless, the compromised surface quality and the recast layer significantly impact the performance of the workpiece machined by EDM. Therefore, for the purpose of enhancing the surface quality and restraining the defects of NMTMCs, this study conducted comparative EDM milling experiments between NMTMCs and Ti6Al4V to analyze the effects of discharge capacitance, charging current, and pulse interval on the surface roughness, recast layer thickness, recast layer uniformity, and surface microcrack density of both materials. The results indicated that machining energy significantly influences workpiece surface quality. Furthermore, comparative experiments exploring the influence of network reinforcement on EDM milling revealed that NMTMCs have a higher melting point, leading to an accumulation phenomenon in low-energy machining where the reinforcement could not be completely removed. The residual reinforcement in the recasting layer had an adsorption effect on molten metal affecting the thermal conductivity and uniformity within the recasting layer. Finally, specific guidelines are put forward for optimizing the material’s surface roughness, recast layer thickness, and uniformity, along with minimizing microcrack density, which attain a processing effect that features a roughness of Ra 0.9 μm, an average recast layer thickness of 6 μm with a range of 8 μm, and a surface microcrack density of 0.08 μm^−1^.

## 1. Introduction

With the ongoing development of metal matrix composites, titanium-based composites have emerged as a promising material, boasting high strength, excellent ductility, remarkable wear resistance, and superior high-temperature durability. These exceptional properties have led to their widespread application in aerospace and advanced military fields [1,2,3]. Unfortunately, research has revealed that the majority of titanium matrix composites currently feature a uniform distribution of reinforcement within the matrix material, leading to subpar mechanical properties and serious ambient temperature brittleness [4]. To tackle this challenge, Huang et al. [5] successfully fabricated a titanium matrix composite featuring a reticulated structure. Employing low-energy ball milling techniques, titanium alloy material was meticulously milled into spheres measuring 220 μm in diameter. Subsequently, a 1–8 micrometer TiB2 powder was carefully prepared and thoroughly mixed with the titanium alloy using a planetary mixer for a duration of eight hours. The homogenous powder blend was then subjected to hot pressing in a vacuum environment at a temperature of 1200 degrees Celsius. The process involved maintaining a pressure of 20 megapascals for a period of 60 min to facilitate an in situ self-propagating reaction, ultimately leading to the successful synthesis of a titanium matrix composite (TiBw/Ti6Al4V) exhibiting a unique network structure. This composite was reinforced with a reticulated distribution of TiB whiskers (TiBw) and employed Ti6Al4V as the matrix material. The unique capsule structure, where the hard phase encompassed the soft phase, enabled the composite to demonstrate superior strength, stiffness, and elastic modulus, while also exhibiting enhanced ductility, wear resistance, and high-temperature durability [6,7,8,9]. Its operational temperature is elevated by 200 °C over Ti6Al4V [10,11,12], and when compared to the commonly used nickel-based superalloys, it boasts a reduced specific gravity [13], thus fulfilling the need for lightweight materials in industry.

However, composite materials pose significant challenges during processing due to their high hardness and brittleness of the reinforcement. When traditional mechanical machining is employed, issues such as severe tool wear, fluctuations in cutting force, elevated cutting temperatures, and difficulties in chip removal often arise [14,15]. Fan et al. [16] conducted an extensive study on the machinability characteristics of the novel Ti-555 high-strength titanium alloy matrix composite during processing. The composite’s inherent high strength and hardness pose significant constraints on machining parameter selection, particularly limiting the cutting speed to a maximum of 45 m/min. Notably, the cutting force exhibits a direct correlation with both cutting depth and feed rate, with cutting depth exerting the most profound influence. Furthermore, the feed rate plays a crucial role in determining the surface roughness of the material, as a higher feed speed not only enhances surface roughness but also intensifies tool wear. In a separate study, Huan et al. [17] explored the intricate relationship between cutting speed and both the surface roughness and tool life of titanium matrix composites during mechanical machining. Their findings revealed that across cutting speeds ranging from 60 m/min to 120 m/min, the surface roughness of the workpiece fluctuated between Ra 0.44 and Ra 0.53 μm. Concurrently, the tool life decreased progressively from approximately 10.5 min at 60 m/min to 5.5 min at 120 m/min, highlighting the need for careful consideration of cutting parameters to optimize both surface quality and tool durability.

To circumvent the various challenges associated with traditional machining of composite materials, scholars have embarked on exploring specialized processing techniques for titanium matrix composites. Among these techniques, electrochemical machining (ECM) stands out as a unique processing method that removes material from the workpiece through electrochemical dissolution of the anode. This technique is particularly advantageous as it can process metallic materials regardless of their hardness, eliminating concerns about tool wear and residual stress [18,19]. ECM technology has gained widespread adoption in the processing of aluminum matrix composites [20,21], magnesium matrix composites [22,23], and metal compound matrix composites [24]. Wang et al. [25] conducted a comprehensive study on the anode dissolution characteristics of two typical g-TiAl alloys in a NaNO_3_ solution. Their experiments encompassed both electrolytic and electrochemical machining, providing a detailed analysis of the observed phenomena. The results revealed that the passivation film is primarily composed of TiO_2_ and Al_2_O_3_, exhibiting a structure that comprises a loose outer layer and a dense inner layer. Additionally, ECM milling experiments were conducted at various feed rates, revealing the complete exposure of the lamellar structure of both alloys and providing insights into the surface quality of these materials. Furthermore, Yue et al. [26] reported on the anode electrolysis behavior of the titanium matrix composite (TiB + TiC)/Ti6Al4V in both 10 wt% NaNO_3_ and NaCl electrolytes. Utilizing techniques such as open-circuit potential testing, potentiodynamic polarization testing, and electrochemical impedance spectroscopy, they discovered that in the NaNO_3_ electrolyte, the passivation film can effectively grow in the pitting region, forming a stable layer that hinders the formation of corrosion pits, thus enhancing corrosion resistance. Interestingly, despite the adhesion of by-products and mechanical losses associated with the NaNO_3_ electrolyte, the current efficiency was found to be higher in the NaCl electrolyte, leading to significantly improved surface quality of the dissolved material.

EDM is a specialized processing technique that leverages pulsed discharges between electrodes to create a discharge channel, releasing substantial heat to erode material from the workpiece. This advanced machining method offers numerous advantages, including the capability to process challenging materials, elimination of macro-scale mechanical forces, and facilitation of high-precision machining operations [27,28]. Due to the challenges associated with machining composite materials, electrical discharge machining technology has become a widely adopted method for processing aluminum matrix composites [29,30], magnesium matrix composites [31,32], and titanium matrix composites [33,34]. Karthikeyan R et al. [35] developed a mathematical model to optimize EDM characteristics in aluminum–silicon carbide particle composites. This model encompassed linear, quadratic, and interaction effects pertaining to material removal rate (MRR), tool wear rate, and surface roughness. Their findings revealed that MRR escalated with increased current but declined with a higher SiC volume percentage and pulse width. Conversely, the tool wear rate escalated with both increased current and SiC volume percentage but diminished with a wider pulse width. Additionally, surface roughness was augmented with escalating current, SiC volume percentage, and pulse duration. To optimize process parameters, the researchers employed equations to minimize tool wear rate (TWR) and surface roughness while maximizing MRR. Sung et al. [36] extensively studied the EDM characteristics of graphene nanoplatelets (GNPs)/Al_2_O_3_ ceramic composites. Their investigation entailed EDM milling experiments that delved into the surface roughness and morphology of the materials across varying discharge energies and concentrations of GNPs. The integration of GNPs into the ceramic composites resulted in distinct changes in grain size, hardness, and crystallinity. Furthermore, the research team analyzed the surface roughness and morphology of the composites across different discharge energy levels. A notable trend emerged: as both the discharge energy and the GNP content increased, the surface roughness escalated. The formation of microcracks and pores on the processed surface was greatly influenced by the fracture toughness, thermal resistance, and thermal expansion rate inherent to the GNP/Al_2_O_3_ ceramic composites. Li et al. [33] embarked on experimental studies to assess the machinability of carbon nanotube-reinforced titanium matrix composites through milling and EDM techniques. Their analysis encompassed surface roughness, hardness, cutting forces, and micromorphology to comprehensively evaluate machinability. Their results underscored the significance of the carbon nanotube reinforcement and in situ formed TiC particles in the Ti matrix in determining the surface quality of milled workpieces. On the contrary, EDM effectively eliminated the recalcitrant TiC particles. Hu et al. [34] delved into the EDM performance of NMTMCs, which comprised a 5% vol. reticulated distribution of TiBw integrated with a Ti6Al4V matrix. They conducted rigorous single-pulse and continuous-pulse experiments across varying capacitance conditions and benchmarked their findings against those obtained from processing Ti6Al4V alloy under identical conditions. Their investigation unpacked the influence of TiBw on the EDM process and introduced a groundbreaking linear absorption model for pulse energy absorption by reinforcement. Furthermore, they explored the impact of discharge capacitance on the material removal rate during EDM milling of both NMTMCs and Ti6Al4V alloy. Building upon Hu’s pioneering research, this study further explores the machining experiments of NMTMCs, aiming to decipher the intricate influence of processing parameters on the surface quality of workpieces. Through meticulous comparative experiments with Ti6Al4V alloy, we seek to elucidate the nuanced effects of reinforcement on the EDM process.

In this paper, a comparative study was conducted on the electrical discharge milling of NMTMCs and Ti6Al4V, aiming to assess the varying impacts of three key electrical parameters—discharge capacitance, charging current, and pulse gap—on the surface quality of the processed workpiece. The focus was particularly on elucidating the effects of incorporating network reinforcements on the electrical discharge machining process. Furthermore, an analysis was undertaken to understand the material removal mechanism following the introduction of these reinforcements. By conducting comparative experiments with a focus on the reinforcement phase, this study offers valuable insights and establishes a foundation for future research endeavors in the field of electrical discharge machining of composite materials.

## 2. Materials and Methods

**Figure 1:**
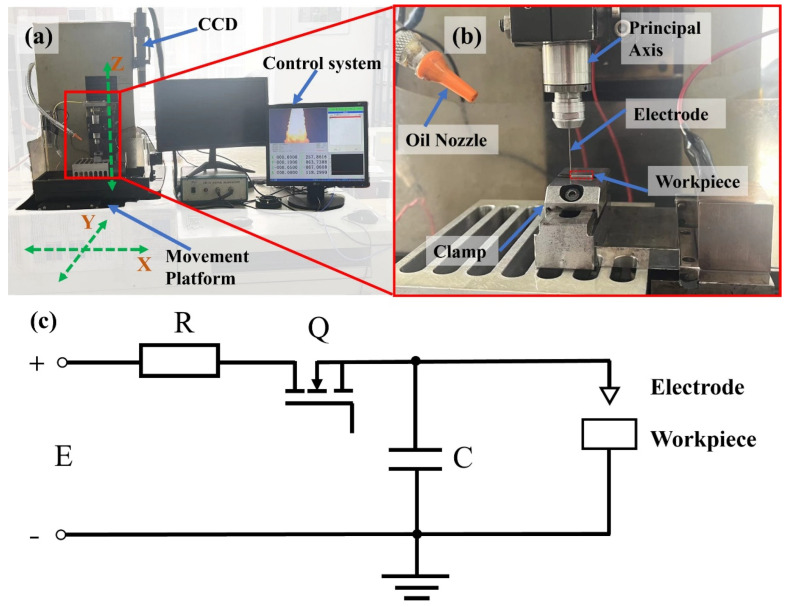
Micro-EDM machine tool: (**a**) machine tool overall structure diagram; (**b**) machine tool local structure diagram; (**c**) machine tool main circuit schematic.

The EDM experiments conducted in this study were performed exclusively on a state-of-the-art three-axis micro-EDM machine, pioneered by Harbin Institute of Technology. This sophisticated machine comprises multiple components, including a transistor-based RC composite pulse power supply, a discharge detection system, a servo feed system, and a working fluid circulation system, as depicted in Figure 1a,b. The transistor-enhanced RC pulse power supply leverages its switching capabilities to precisely adjust pulse width and pulse interval, offering unparalleled control over the EDM process. The intricate main circuit diagram is illustrated in Figure 1c. During operation, the user can configure the pulse width and pulse interval according to specific machining requirements. Initially, the switch Q is activated, enabling the DC power supply E to charge the discharge capacitance C through resistance R. Once the preset pulse width is reached, the switch Q is deactivated, initiating the pulse interval. As the tool electrode approaches the workpiece to a critical distance, a breakdown discharge occurs, resulting in a rapid voltage drop and the generation of a substantial peak current. This intense discharge releases a considerable amount of heat, effectively eroding the workpiece material. The cycle repeats as the next pulse width begins, with the switch Q being reactivated. The machine boasts exceptional precision, offering X, Y, Z feed directions with a positioning accuracy of up to 0.1 μm. Real-time monitoring of the electrode’s morphology is achieved through the integration of a CCD camera. Post-processing analysis is conducted using laser confocal microscope (OLS3000, Olympus, Tokyo, Japan) to accurately assess surface roughness, while the microstructure of the processed samples is meticulously observed using a FESEM (SU8010, Hitachi, Tokyo, Japan) electron scanning microscope.

**Figure 2:**
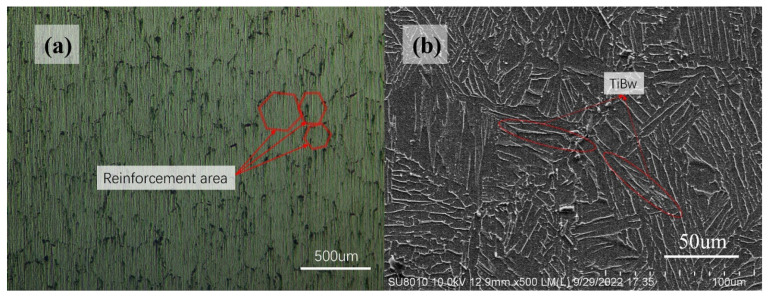
(**a**) Optical micrograph of the unetched NMTMCs; (**b**) SEM image of the etched NMTMCs.

The material utilized in this study was a reticulated titanium matrix composite, pioneered by the Huang Lujun team [5]. The composite’s backbone comprises Ti6Al4V alloy, reinforced with 5% volume fraction of TiBw whiskers. The detailed chemical composition of this material is outlined in Table 1. To facilitate the observation of the reinforcement, the reticulated titanium matrix composite samples were meticulously ground using sandpapers with grit sizes ranging from P200 to P2000. Subsequently, a polishing treatment was applied, employing a Cr_2_O_3_ polishing agent with an average particle size of 1.5 μm. The optical microscope image of the polished surface, depicted in Figure 2a, clearly reveals the concentrated distribution of TiBw in the reticulated reinforcement. The metal matrix is segmented into irregular polygons, exhibiting a characteristic size of 180–220 μm. To further enhance the visibility of the reinforcement, the polished surface was etched using Kroll’s reagent, a mixture of 5% vol. HF, 15% vol. HNO_3_, and 80% H_2_O, for a duration of 10 s. The resulting SEM image, presented in Figure 2b, offers a more intricate view of the reinforcement on the material’s surface.

Through preliminary experimental research, it was discovered that the surface quality of the processed workpiece is primarily influenced by factors such as pulse width, pulse interval, discharge capacitance, and charging current. Differences in pulse width and charging current can lead to variations in the voltage $u_{c}$ of the discharge capacitance after the pulse width ends. To maintain a consistent capacitance voltage $u_{c}$, an orthogonal experiment was designed, considering pulse interval, discharge capacitance, and charging current. Prior to the experiment, an open-circuit voltage of 100 V was preset to charge the capacitance voltage $u_{c}$ to 100 V. Subsequently, the pulse width time was determined based on the charging current. Given that the RC transistor composite pulse power supply leverages the switching characteristics of the transistor, the circuit depicted in Figure 1c can be approximated as an RC circuit during the pulse width. In such an RC circuit, the capacitance voltage follows a specific mathematical relationship, as outlined in Equation (1):

(1) $$u_{c}=E*(1-e^{-\frac{t}{\tau }})$$

where $u_{c}$ represents the capacitance voltage (in volts, V); *E* represents the open-circuit voltage (in volts, V); *t* represents the charging time (in microseconds, μs); *τ* represents the time constant (in microseconds, μs). In this context, the time constant *τ* is related to the capacitance and the charging current, which can be determined by referring to a table based on the electrical parameters of the machine tool. Due to the decay characteristics of the negative exponential function, the time for the capacitance voltage to charge up to the open-circuit voltage can be approximately calculated, as shown in Equation (2):

(2) t=5τ

In this experiment, a micro-electrode was first prepared using electrode grinding technology, followed by conducting micro-milling experiments on the workpiece surface. The specific micro-EDM milling process parameters are outlined in Table 2.

**Figure 3:**
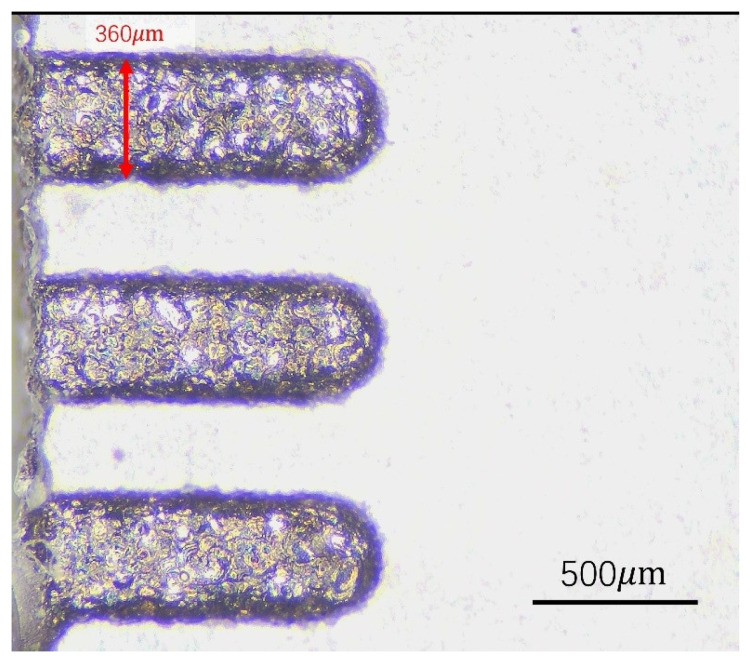
Micro-EDM milling profile map.

Based on the preliminary experimental results, a three-factor, three-level orthogonal experiment was designed in this study, considering the discharge capacitance, charging current, and pulse interval as the factors. Each experimental group was repeated three times, and the experimental design is summarized in Table 3. Using a 300-micrometer diameter electrode, a groove with a depth of 100 μm and a length of 1000 μm was milled on the surface of the workpiece, as shown in Figure 3.

After processing, the roughness, average thickness, and uniformity of the recast layer, as well as the density of surface microcracks, were measured on the workpiece surface. These measurements were used to analyze the surface quality characteristics of NMTMCs during micro-EDM milling and to compare them with those obtained from Ti6Al4V micro-EDM milling experiments. The aim was to investigate the influence of network reinforcement on the processing of the material.

Finally, the optimized processing parameters for the surface of NMTMCs were derived from the orthogonal experiments, followed by a single-factor verification experiment to assess the surface roughness, recast layer thickness, range of recast layer thickness, and density of surface microcracks of NMTMCs after process optimization.

## 3. Results and Discussion

### 3.1. Surface Roughness

The surface resulting from EDM is distinct from that achieved through traditional machining methods, consisting of numerous irregular and nondirectional pits that confer superior surface lubricity and wear resistance. Surface roughness is typically characterized by the arithmetic mean deviation of micro-irregularities (Ra) and the maximum height of these micro-irregularities (Rmax). In the context of EDM, various factors influence surface roughness, including the materials of the electrode and workpiece, single-pulse energy, working fluid, and pulse interval. Notably, single-pulse energy exerts the most profound influence on surface roughness. In this experiment, we employed the arithmetic mean deviation Ra as a metric to quantify the surface roughness of the processed workpiece, as detailed in Equation (3):

(3) $$Ra=\frac{1}{l}\int_{0}^{l}\left|Z(x)\right|dx$$

where *Ra* represents the arithmetic mean deviation of micro-irregularities (in micron, μm); *l* represents the sampling length (in micron, μm); *Z*(*x*) represents the function representing the difference between the micro-profile height of the workpiece and the average height. Roughness sampling length (lr) is 0.8 mm, roughness evaluation length (ln) is 4 mm, and cut off ($\lambda_{c}$) is 1/10.

**Figure 4:**
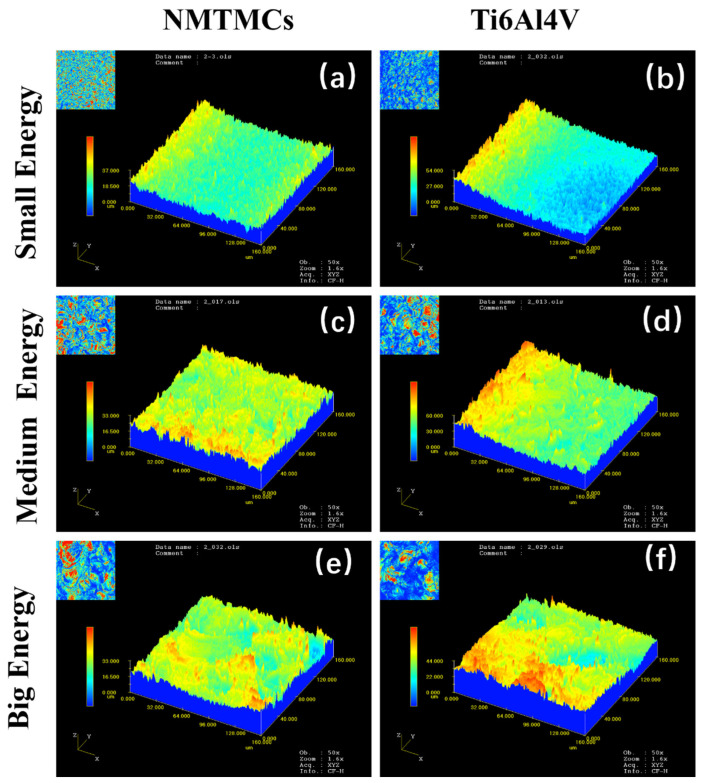
Surface morphologies of NMTMCs and Ti6Al4V observed through laser confocal microscopy under different processing energies. (**a**) surface morphology of small energy processing with NMTMCs (**b**) surface morphology of small energy processing with Ti6Al4V (**c**) surface morphology of medium energy processing with NMTMCs (**d**) surface morphology of medium energy processing with Ti6Al4V (**e**) surface morphology of big energy processing with NMTMCs (**f**) surface morphology of big energy processing with Ti6Al4V.

Utilizing the OLS3000 laser confocal microscope, we examined the surface morphology of the processed workpiece and fitted its roughness accordingly. Figure 4 demonstrates the surface morphologies of NMTMCs and Ti6Al4V captured by laser confocal microscopy under varying processing energies. The surfaces exhibit a pattern of individual discharge craters, and as the discharge energy intensifies, the diameter of these craters correspondingly increases. Furthermore, under identical processing parameters, the degree of undulation in the discharge craters on the surface of NMTMC workpieces is noticeably lower than that observed on Ti6Al4V workpieces.

**Figure 5:**
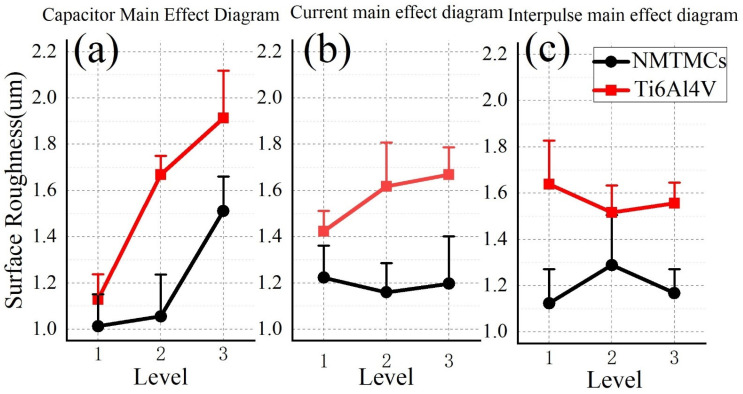
Main effect diagram of surface roughness between NMTMCs and Ti6Al4V: (**a**) capacitance; (**b**) current; (**c**) pulse interval.

Figure 5 presents the main effect plots of surface roughness for NMTMCs and Ti6Al4V. As can be seen from Figure 5a, as the discharge capacitance increases, the surface roughness of both materials also increases. This is because a larger discharge capacitance results in greater discharge energy and material removal rate, leading to larger discharge craters and consequently higher surface roughness. However, the growth rate of surface roughness for NMTMCs increases with the increase in discharge capacitance, while the growth rate for Ti6Al4V does not increase significantly. Additionally, under the same processing parameters, the surface roughness of NMTMCs is lower than that of Ti6Al4V. This can be attributed to the better high-temperature durability of NMTMCs compared to Ti6Al4V under the same conditions [9], as NMTMCs have a higher melting point than Ti6Al4V. When processed with low energy, NMTMC materials are mainly removed through flushing after melting, resulting in smaller discharge craters and lower surface roughness. As the processing energy increases, material removal shifts to vaporization, leading to a significantly higher material removal rate and an increased growth rate of surface roughness. On the other hand, Ti6Al4V has a lower melting point, and its material removal is primarily through vaporization, even at low energy levels, resulting in less variation in the growth rate of surface roughness. Furthermore, due to the difference in melting points, the material removal rate of Ti6Al4V is higher than that of NMTMCs under the same processing parameters, explaining the generally higher surface roughness of Ti6Al4V workpieces.

Figure 5b indicates that the surface roughness of both materials does not change significantly with the increase in charging current. This is because the charging current does not directly affect the discharge energy. When the voltage across the discharge capacitance is constant, a higher charging current results in a shorter pulse width, which does not affect the surface roughness of the processed workpiece. From Figure 5c, it can be observed that as the pulse interval increases, the surface roughness of NMTMC workpieces first increases and then decreases, while the change in surface roughness for Ti6Al4V workpieces is not significant. Since the melting point of NMTMCs is higher than that of Ti6Al4V, it is speculated that when the pulse interval is too short, the cooling time for NMTMC workpieces is insufficient, leading to partial liquid metal resolidified on the surface of the workpiece, resulting in a low material removal rate and low surface roughness. As the pulse interval increases, some of the liquid metal is removed by the working fluid, while some of it solidifies on the workpiece surface, resulting in a higher material removal rate in the center of the discharge craters and a lower rate at the edges, leading to increased surface roughness. With further increases in the pulse interval, the molten liquid metal is removed more efficiently, resulting in a surface roughness that is slightly higher than that obtained with a short pulse interval but lower than that with a medium pulse interval. On the other hand, Ti6Al4V has a lower melting point and can cool sufficiently even with short pulse intervals, making the effect of pulse interval changes on the surface roughness of Ti6Al4V workpieces less significant.

The mean response table for the surface roughness of NMTMCs is summarized in Table 4. As evident from the table, the discharge capacitance exhibits the most profound influence on the surface roughness of the workpiece during processing, followed closely by the pulse interval. Conversely, the impact of charging current on surface roughness appears to be minimal and nearly negligible.

The mean response table for the surface roughness of Ti6Al4V is outlined in Table 5. Notably, the table reveals that the discharge capacitance exerts the most profound influence on the surface roughness of the workpiece during processing. This is followed by the charging current and, subsequently, the pulse interval. A comparison between Table 4 and Table 5 underscores an interesting observation: under comparable conditions, the surface roughness values of Ti6Al4V workpieces tend to be slightly higher than those of NMTMCs. This finding aligns with the earlier analysis, further validating the trends observed.

In summary, the micro-EDM milling surface roughness of NMTMC workpieces can be optimized by adopting a capacitance range of 10–100 kpF and pulse intervals of either 1 μs or 40 μs.

### 3.2. Average Thickness and Uniformity of the Recast Layer

During the electric discharge machining process, when the electrode and workpiece are positioned close enough, a discharge breakdown occurs, resulting in instantaneous high temperatures. Subsequently, the molten metal material rapidly cools and solidifies under the cooling action of the working fluid, forming a recast layer. Unlike the metal matrix, the recast layer exhibits a dendritic quenched casting structure composed of martensite, a significant amount of fine-grained residual austenite, and certain carbides. However, due to the random and inhomogeneous nature of discharges, the thickness of the recast layer may vary even with identical processing parameters. To accurately represent the thickness of the recast layer, the average thickness is employed, as demonstrated in Equation (4):

(4) $$h=\frac{1}{2n}\sum_{i=1}^{n}\left(h_{{max}_{n}}+h_{{min}_{n}}\right)$$

where *h* represents the average thickness of the recast layer (in micron, μm); *n* represents the number of sampling times; $h_{{max}_{n}}$ represents the maximum thickness of the recast layer in the nth photomicrograph (in micron, μm); $h_{{min}_{n}}$ represents the minimum thickness of the recast layer in the nth photomicrograph (in micron, μm).

To accurately assess the uniformity of the recast layer, considering its inherent inhomogeneity, multiple samplings are conducted. The maximum and minimum thicknesses of the recast layer across all photomicrographs are determined, and the range between these values is calculated. This range serves as a metric to quantify the degree of uniformity in the recast layer, as outlined in Equation (5):

(5) $$R=h_{max}-h_{min}$$

where *R* represents the range of thicknesses of the recast layer obtained through sampling (in micron, μm); $h_{max}$ represents the maximum thickness of the recast layer across all photomicrographs obtained through sampling (in micron, μm); $h_{min}$ represents the minimum thickness of the recast layer across all photomicrographs obtained through sampling (in micron, μm).

**Figure 6:**
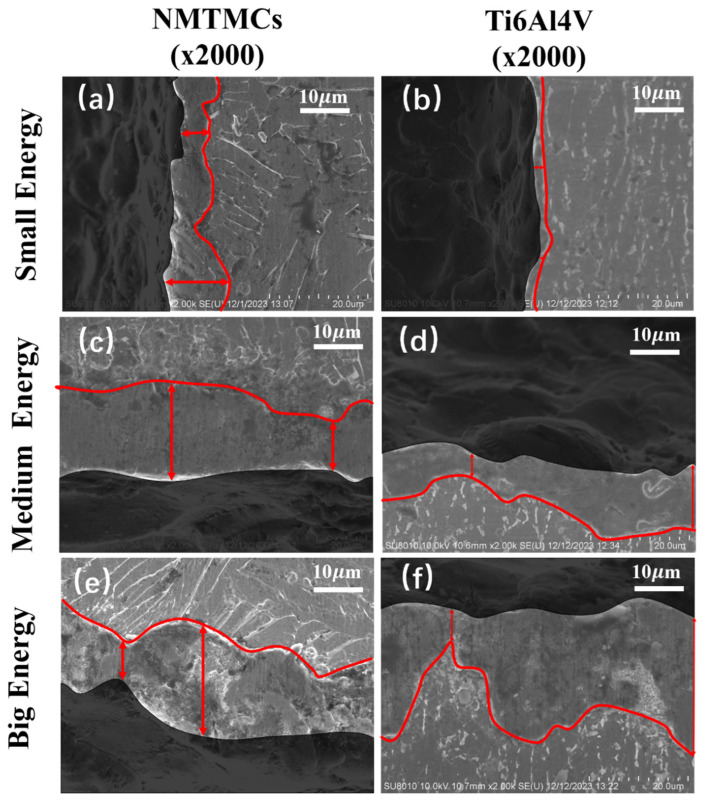
SEM of NMTMCs and Ti6Al4V recast layers under different energy levels. (**a**) recast layer formed by small energy processing with NMTMCs (**b**) recast layer formed by small energy processing with Ti6Al4V (**c**) recast layer formed by medium energy processing with NMTMCs (**d**) recast layer formed by medium energy processing with Ti6Al4V (**e**) recast layer formed by big energy processing with NMTMCs (**f**) recast layer formed by big energy processing with Ti6Al4V.

EDM invariably gives rise to a recast layer, characterized by the presence of numerous defects like microcracks and micropores on its surface. In the case of composite materials, the swift cooling and solidification processes following the thermal removal of material alter the form of the reinforcement within the recast layer [37]. Consequently, the surface properties of the recast layer differ significantly from those of the metal matrix. It is, therefore, of utmost importance to delve into the effects of various electrical machining parameters on both the thickness and the uniformity of the recast layer. By leveraging the distinct properties of the recast layer and the metal matrix, the workpiece surface can be etched, enabling clear observation of the recast layer under an electron microscope for varying processing energies, and the red arrows indicate the maximum and minimum widths of the recast layer, as exemplified in Figure 6.

**Figure 7:**
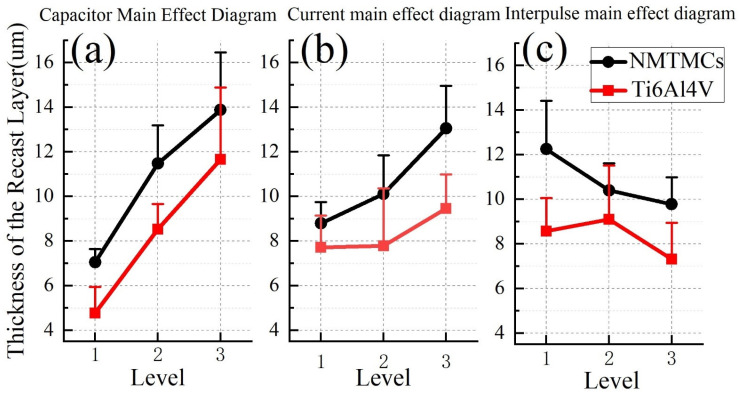
Main effect diagram of average thickness of the recast layer between NMTMCs and Ti6Al4V: (**a**) capacitance; (**b**) current; (**c**) pulse interval.

Based on the scale bar, the maximum and minimum thicknesses of the recast layer within each electron microscopy image’s field of view are determined, and the average of these two values is calculated as the average thickness of the recast layer. Figure 7a presents the capacitance main effect plot comparing the average thickness of the recast layers for both materials. As the capacitance of the discharge capacitance increases, leading to a higher discharge energy, the material removal rate also increases. This increased removal results in a thicker recast layer due to the increased cooling and solidification processes. However, under identical processing conditions, the NMTMCs exhibit a thicker average recast layer compared to Ti6Al4V. This can be attributed to the adsorption of molten liquid metal by the reinforcement in NMTMCs. When processed with low energy, the NMTMC workpiece surface retains a higher concentration of residual reinforcement, enhancing its ability to adsorb the molten metal. Consequently, the average thickness of the recast layer for NMTMCs is 1.48 times thicker than that of Ti6Al4V under the same conditions. As the discharge energy is increased, the residual reinforcement on the NMTMC workpiece surface decreases, weakening its adsorption effect on the molten metal. Under high-energy processing conditions, the average thickness of the recast layer for NMTMCs decreases, but it remains 1.19 times thicker than that of Ti6Al4V under comparable conditions.

As depicted in Figure 7b, the average thickness of the recast layer in NMTMCs exhibits a positive correlation with the charging current. As the charging current intensifies, the pulse width narrows, subsequently reducing the overall pulse duration. During micro-EDM milling, the electrode’s feed rate remains relatively sluggish. Consequently, under higher charging currents, the discharge distance diminishes during each discharge event, promoting the accumulation of the recast layer and subsequently increasing its average thickness. Conversely, the average thickness of the recast layer in Ti6Al4V remains less sensitive to charging current variations. Given that Ti6Al4V possesses a lower melting point than NMTMCs, its machining process is smoother, experiencing fewer instances of short-circuiting and retractions. This characteristic minimizes the accumulation of the recast layer during machining, thereby mitigating the impact of charging current on its thickness.

Figure 7c reveals that the pulse interval plays a pivotal role in determining the duration between pulses. An increase in the pulse interval elongates the overall pulse duration, making it less probable for the recast layer in NMTMCs to accumulate, thus resulting in a thinner average thickness. Similarly, the average thickness of the recast layer in Ti6Al4V remains relatively unaffected by variations in the pulse interval.

The mean response table summarized in Table 6 illustrates the varying impacts on the average thickness of the recast layer in NMTMCs. Among the factors, the discharge capacitance exerts the most prominent influence, closely followed by the charging current, and lastly, the pulse interval.

The mean response table outlined in Table 7 reveals the varying degrees of influence on the average thickness of the recast layer in Ti6Al4V. Notably, the discharge capacitance emerges as the most significant factor, followed closely by the pulse interval, and ultimately, the charging current.

In summary, the micro-EDM milling of the NMTMC workpiece can be optimized by employing a 10 kpF capacitance, a charging current of 0.11 A, and a pulse interval of 40 μs, thereby minimizing the thickness of the recast layer.

**Figure 8:**
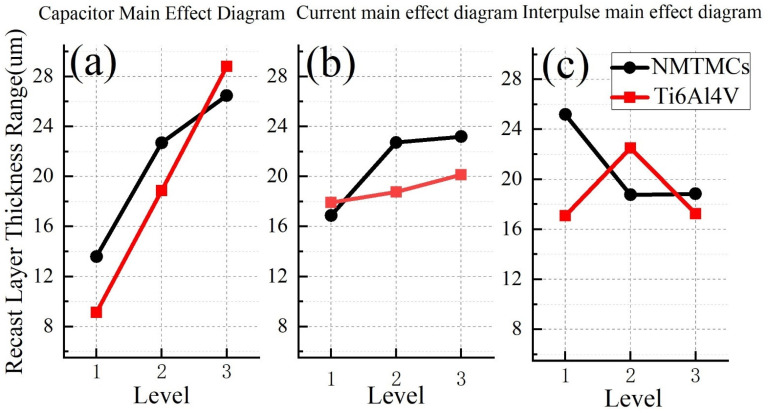
Main effect diagrams of recast layer thickness range between NMTMCs and Ti6Al4V: (**a**) capacitance; (**b**) current; (**c**) pulse interval.

The uniformity of the recast layer serves as a critical indicator of its overall properties. The inherent randomness of EDM processes often results in non-uniform thickness within the recast layer. Consequently, we employ the recast layer’s thickness range as a metric to assess its uniformity. Figure 8 presents the main effect diagram for the recast layer’s thickness range of both materials. As evident in Figure 8a, an increase in discharge capacitance leads to a widening range of thickness variations in both NMTMCs and Ti6Al4V. This is attributed to the higher discharge energy associated with a larger discharge capacitance, causing random discharges from the tool electrode and, in turn, enhancing thickness variations. However, under conditions of low machining energy, NMTMCs exhibit a greater range of thickness variations compared to Ti6Al4V. This is due to the uneven exposure of reinforcement on the NMTMC surface, which attracts liquid metal and contributes to the increased variations. As the machining energy rises to a certain threshold, most reinforcement on the workpiece surface is removed, resulting in comparable ranges of thickness variations in both materials.

Figure 8b,c illustrate that in NMTMCs, the range of thickness variations initially rises with an increase in charging current but then plateaus. Similarly, as the pulse interval widens, the range of thickness variations decreases initially and then stabilizes. Since charging current and pulse width are inversely related, it can be deduced that the overall pulse duration significantly impacts the range of thickness variations in the workpiece’s recast layer. Limited tool electrode feed rates result in closer discharge distances on the workpiece during shorter pulse durations, leading to repeated discharges and greater thickness variations. Conversely, as the pulse duration extends, discharge distances on the workpiece increase, eliminating repeated discharges and stabilizing the range of thickness variations at a lower level. Conversely, Ti6Al4V, with its lower melting point, is less susceptible to the accumulation of the recast layer. Consequently, the range of thickness variations in its recast layer is less influenced by the overall pulse duration, rendering the effects of charging current and pulse interval insignificant.

The mean response table presented in Table 8 illustrates the variance in the thickness of the recast layer for NMTMCs. Notably, the discharge capacitance exhibits the most prominent influence on this variance, whereas the charging current and pulse interval exert comparatively lesser effects.

The mean response table for the variance in recast layer thickness of Ti6Al4V is detailed in Table 9. Notably, the discharge capacitance stands out as the most influential factor, whereas the charging current and pulse interval exert relatively minor impacts.

In summary, the inhomogeneity of the recast layer during the micro-EDM milling process of the NMTMC workpiece can be effectively minimized by utilizing a 10 kpF capacitance, a charging current of 0.11 A, and a pulse interval of 40 μs.

### 3.3. Surface Microcrack Density

When the surface of the workpiece is exposed to electric spark discharge, it experiences instantaneous high-temperature melting and rapid cooling and solidification under the influence of the working fluid. This process generates residual tensile stress, leading to the formation of microcracks on the surface, which in turn compromises the surface properties of the workpiece. To quantify the extent of these microcracks, we utilized the surface microcrack density as a metric, as outlined in Equation (6):

(6) $$\rho =\frac{L}{S}$$

where *ρ* represents the surface microcrack density (in per micron, μm^−1^); *L* represents the total length of cracks in the sampled electron micrograph (in micron, μm); *S* represents the area of the sampled electron micrograph (in square micron, μm^2^).

**Figure 9:**
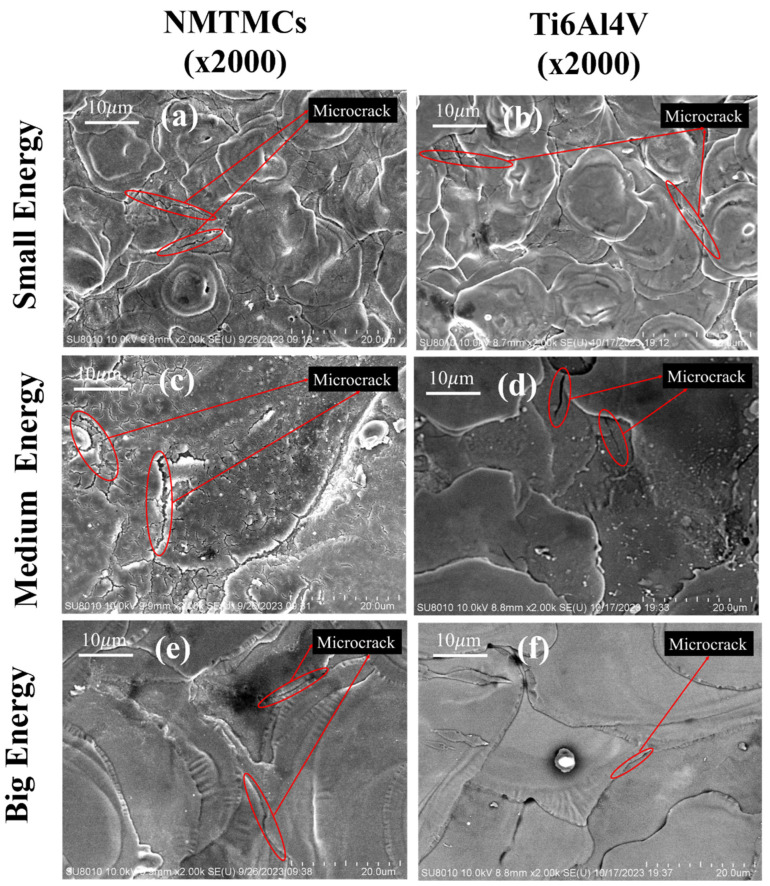
SEM of surface microcracks on NMTMCs and Ti6Al4V processed under different energy levels. (**a**) surface microcracks formed by small energy processing with NMTMCs (**b**) surface microcracks formed by small energy processing with Ti6Al4V (**c**) surface microcracks formed by medium energy processing with NMTMCs (**d**) surface microcracks formed by medium energy processing with Ti6Al4V (**e**) surface microcracks formed by big energy processing with NMTMCs (**f**) surface microcracks formed by big energy processing with Ti6Al4V.

The microcracks present on the machined surface of the workpiece, as depicted in Figure 9, have a profound effect on its surface quality, leading to a decrease in fatigue strength [38]. Consequently, studying the impact of machining electrical parameters on the density of these surface microcracks holds significant importance.

**Figure 10:**
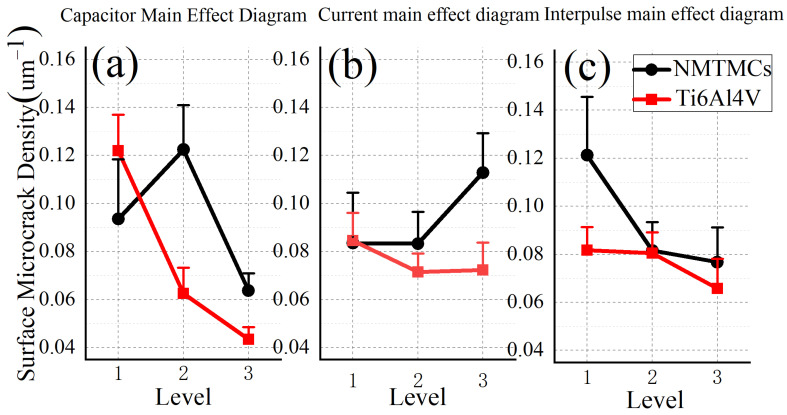
Main effect diagram of surface microcrack density between NMTMCs and Ti6Al4V: (**a**) capacitance; (**b**) current; (**c**) pulse interval.

As depicted in Figure 10a, the surface microcrack density of NMTMCs processed via EDM initially surges and subsequently tapers off with an increase in discharge capacitance. This trend can be attributed to the transition in material removal mechanisms. During medium- to low-energy processing, the primary mechanism of material removal involves melting and flushing with liquid. Initially, at lower energy levels, a thinner recast layer is formed. However, as the discharge capacitance gradually increases, the resolidified liquid metal encapsulates the reinforcing phase, leading to a marked decline in the material’s thermal conductivity. Consequently, this gives rise to substantial residual tensile stress and a corresponding elevation in the density of surface microcracks. However, as the discharge capacitance reaches a critical threshold, material removal shifts towards vaporization, causing a decrease in the reinforcement content within the recast layer [31], which leads to the formation of a thicker and more uniform recast layer. This shift results in more uniform heat conduction, decreased residual stress, and, ultimately, a reduction in surface microcrack density. On the other hand, the surface microcrack density of Ti6Al4V exhibits a decreasing trend with an increase in discharge capacitance. As illustrated in Figure 7a, a larger discharge capacitance correlates with a thicker average recast layer for Ti6Al4V, promoting more uniform heat conduction. This, in turn, results in smaller residual tensile stress and, consequently, a lower surface microcrack density.

Figure 10b,c reveal that the surface microcrack density of NMTMCs remains stable initially and then rises with an increase in charging current. Conversely, it decreases and subsequently plateaus with an increase in pulse interval. This behavior can be explained by the inhomogeneous distribution of the reinforcement within the recast layer, leading to poor heat conduction. This inhomogeneity also accounts for the higher surface microcrack density observed in NMTMCs compared to Ti6Al4V. The unevenness of the NMTMC recast layer renders its surface microcrack density highly sensitive to cooling time. High charging currents result in narrow pulse widths, and a combination of narrow pulse widths and short pulse intervals can lead to inadequate cooling time between discharges. This insufficient cooling time contributes to a higher surface microcrack density in NMTMCs. However, once the pulse time exceeds a certain threshold, the surface microcrack density remains unaffected. In contrast, the surface microcrack density of Ti6Al4V is less influenced by charging current and pulse interval. The absence of reinforcement in Ti6Al4V results in a more uniform recast layer and superior heat conduction properties, minimizing the sensitivity to cooling time variations.

The mean response table for the surface microcrack density of NMTMCs is outlined in Table 10. According to the table, the discharge capacitance exerts the most prominent influence on the surface microcrack density of the workpiece, followed closely by the pulse interval. Conversely, the charging current has the least notable impact on the surface microcrack density of the workpiece.

The mean response table for the surface microcrack density of Ti6Al4V is clearly delineated in Table 11. According to the tabulated data, the discharge capacitance stands out as the most notable factor influencing the surface microcrack density of the workpiece. In contrast, both the charging current and pulse interval exert comparatively less significant impacts on the surface microcrack density of the workpiece.

In summary, the micro-EDM milling process of the NMTMC workpiece can be optimized to minimize surface microcrack defects by employing either a 10 kpF or 200 kpF capacitance, a charging current of 0.11 A, and a pulse interval of either 20 μs or 40 μs.

### 3.4. Single-Factor Verification Experiment

For NMTMC workpieces, the optimal electrical parameters for micro-EDM milling processing include a 10 kpF capacitance, a charging current of 0.11 A, and a pulse interval of 40 μs. On this foundation, three sets of single-factor verification experiments were meticulously designed to ascertain the most suitable process parameters for NMTMCs. During each experiment, only one factor was varied while the remaining two were maintained at their optimal settings, as shown in Table 12, Table 13 and Table 14.

**Figure 11:**
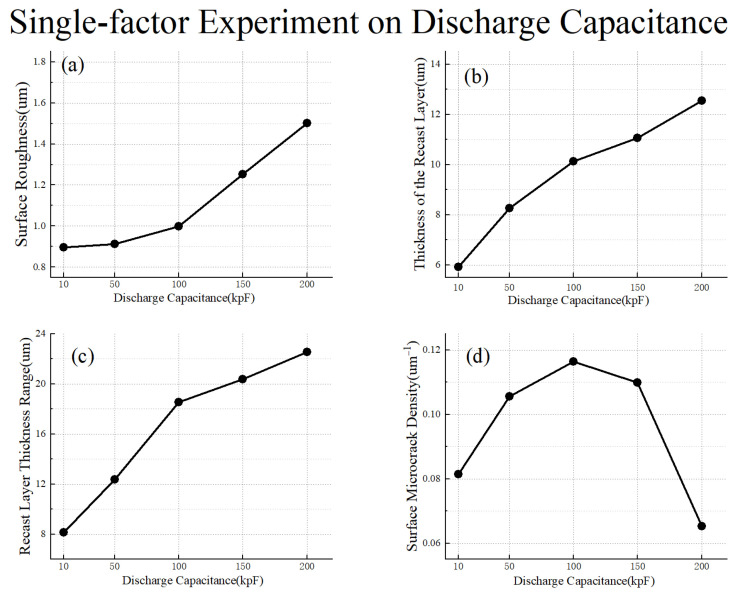
Single-factor experiment on discharge capacitance: (**a**) surface roughness; (**b**) thickness of the recast layer; (**c**) recast layer thickness range; (**d**) surface microcrack density.

As shown in Figure 11, with the increase in discharge capacitance, the surface roughness, the thickness of the recast layer, and the recast layer thickness range all increase accordingly. Additionally, the growth rate of surface roughness also increases as the discharge capacitance increases, which is consistent with the previously analyzed conclusion. Simultaneously, as the discharge capacitance increases, the density of surface microcracks exhibits a trend of initial increase followed by a decrease. Notably, the density remains consistently high within the range of 50 kpF to 150 kpF for the discharge capacitance. Based on these observations, we can deduce that when the discharge capacitance is minimal, the recast layer assumes a thinner profile, resulting in average thermal conductivity. However, as the discharge capacitance increases, a considerable amount of reinforced phase is incorporated within the recast layer, leading to a precipitous decline in thermal conductivity and a corresponding surge in the density of surface microcracks. Conversely, once the discharge capacitance attains a critical threshold, the reinforced phase within the recast layer is effectively eliminated, yielding a thicker and more uniform recast layer that exhibits superior thermal conductivity and a reduced density of surface microcracks.

**Figure 12:**
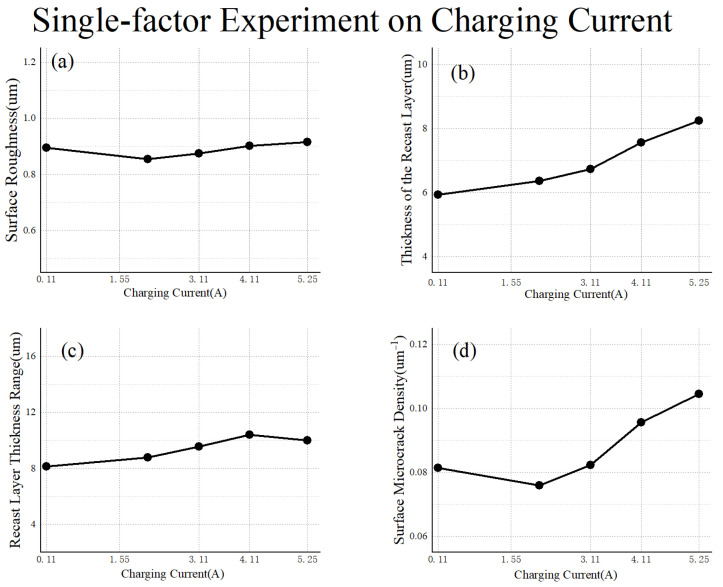
Single-factor experiment on charging current: (**a**) surface roughness; (**b**) thickness of the recast layer; (**c**) recast layer thickness range; (**d**) surface microcrack density.

As shown in Figure 12, under conditions of low energy and large pulse intervals, the surface roughness and the recast layer thickness range exhibit minimal changes with the increase in charging capacitance. With the growth of charging capacitance, the thickness of the recast layer and the density of surface microcracks initially remain relatively stable, followed by a slight increase. This can be explained by the limitations imposed by the conditions of low energy and large pulse intervals.

**Figure 13:**
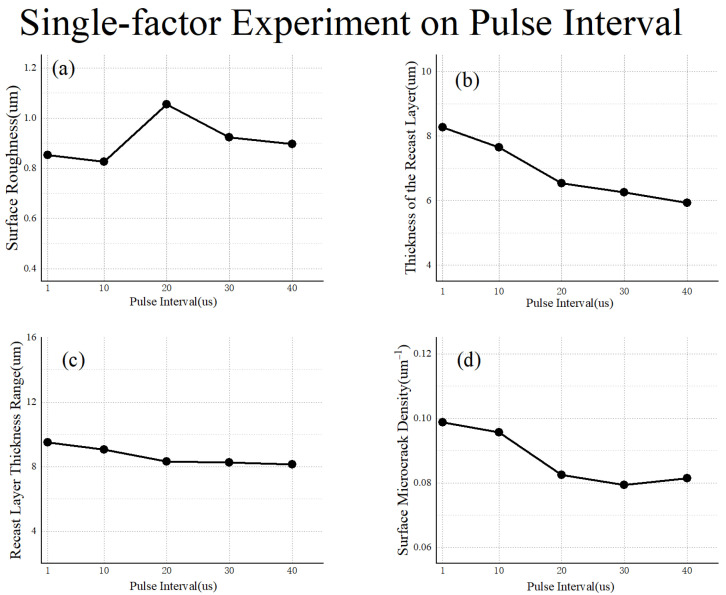
Single-factor experiment on pulse interval: (**a**) surface roughness; (**b**) thickness of the recast layer; (**c**) recast layer thickness range; (**d**) surface microcrack density.

Similarly, given the low energy and small charging current, the pulse interval exerts a minor influence on the surface roughness and the recast layer thickness range. As the pulse interval widens, the thickness of the recast layer and the density of surface microcracks decrease, aligning with prior observations. However, the magnitude of these variations remains relatively insignificant, as depicted in Figure 13.

In conclusion, the optimal process parameters for micro-EDM milling of NMTMCs comprise a discharge capacitance of 10 kpF, a charging current of 0.11 A, and a pulse interval of 40 μs, which attain a processing effect that features a roughness of Ra 0.9 μm, an average recast layer thickness of 6 μm with a range of 8 μm, and a surface microcrack density of 0.08 μm^−1^.

## 4. Conclusions

This paper delves into the impact of three electrical parameters—discharge capacitance, charging current, and pulse interval—on the surface roughness, average thickness, and uniformity of the recast layer, as well as the surface microcrack density of NMTMCs during micro-EDM milling. Additionally, a comparative analysis with Ti6Al4V is conducted to explore the influence of NMTMCs reinforcements on the milling process. The key findings from this chapter are summarized as follows:

- These three electrical parameters have varying degrees of influence on the surface quality properties of NMTMC workpieces, and in general, the discharge capacitance has the most significant impact on all aspects of the recast layer.
- NMTMCs boast superior high-temperature durability, attributed to their higher melting point compared to Ti6Al4V. This characteristic results in the accumulation of recast layers during low-energy processing.
- During low-energy processing of NMTMCs, reinforcement tends to persist on the workpiece surface, exerting an adsorbing effect on molten metal. This, in turn, affects the uniformity and thermal conductivity of the recast layer.
- For NMTMC workpieces, the optimal electrical parameters for micro-EDM milling processing include a 10 kpF capacitance, a charging current of 0.11 A, and a pulse interval of 40 μs. These parameters can be used to optimize surface roughness, thickness, and inhomogeneity of the recast layer, as well as surface microcrack defects, which attain a processing effect that features a roughness of Ra 0.9 μm, an average recast layer thickness of 6 μm with a range of 8 μm, and a surface microcrack density of 0.08 μm^−1^. Therefore, this approach provides valuable guidance for the micro-EDM milling of NMTMCs.
- This paper presents a comprehensive study on the characteristics of surface roughness, recast layer, and surface microcrack density of NMTMCs during EDM. The findings reveal that while certain defects may exist on the workpiece surface, these can be effectively mitigated by optimizing the EDM parameters. Furthermore, through comparative experiments involving composite materials and their matrix counterparts, the article provides a deeper understanding of the influence of reinforcements on material machinability, elucidating their processing mechanisms and offering guidelines for exploring EDM techniques in composite materials.
- After EDM of the workpiece, there are inevitably some defects on the surface. However, we can adopt electrochemical machining methods to further refine the processing based on electrical discharge machining. This subsequent processing can effectively remove the recast layer, thereby achieving perfect surface quality for the workpiece.

## Figures and Tables

**Table 1:** Chemical composition of NMTMCs and Ti6Al4V.

Materials	Al	V	B	Fe	Si	O	C	N	H	Ti
NMTMCs	6.22	3.99	0.97	0.40	0.024	0.15	0.016	0.010	0.004	Bal.
Ti6Al4V	6.42	4.12	-	0.18	0.024	0.12	0.013	0.011	0.004	Bal.

**Table 2:** Orthogonal experimental parameters for micro-EDM milling.

Parameter	Value
Electrode Diameter (μm)	300
Open-Circuit Voltage (V)	100
Electrode Material	Tungsten
Working Fluid	Kerosene
Polarity	Workpiece (+)
Machining Material	NMTMCs/Ti6Al4V

**Table 3:** Orthogonal experimental factor and level table.

Factor/Level	Discharge Capacitance (kpF)	Charging Current (A)	Pulse Interval (μs)
1	10	0.11	1
2	100	3.11	20
3	200	5.25	40

**Table 4:** Mean response table for surface roughness of NMTMCs.

Level	Capacitance	Current	Pulse Interval
1	1.013	1.223	1.124
2	1.056	1.160	1.289
3	1.511	1.197	1.168
Delta	0.499	0.063	0.165
Rank	1	3	2

**Table 5:** Mean response table for surface roughness of Ti6Al4V.

Level	Capacitance	Current	Pulse Interval
1	1.129	1.424	1.639
2	1.669	1.618	1.516
3	1.913	1.669	1.556
Delta	0.784	0.245	0.123
Rank	1	2	3

**Table 6:** Mean response table for average thickness of the recast layer of NMTMCs.

Level	Capacitance	Current	Pulse Interval
1	7.047	8.792	12.243
2	11.482	10.116	10.392
3	13.881	13.052	9.774
Delta	6.834	4.710	2.469
Rank	1	2	3

**Table 7:** Mean response table for average thickness of the recast layer of Ti6Al4V.

Level	Capacitance	Current	Pulse Interval
1	4.776	7.713	8.562
2	8.531	7.783	9.094
3	11.657	9.467	7.307
Delta	6.881	1.753	1.788
Rank	1	3	2

**Table 8:** Mean response table for the recast layer thickness range of NMTMCs.

Level	Capacitance	Current	Pulse Interval
1	13.59	16.87	25.18
2	22.70	22.72	18.76
3	26.48	23.18	18.83
Delta	12.89	6.32	6.42
Rank	1	3	2

**Table 9:** Mean response table for the recast layer thickness range of Ti6Al4V.

Level	Capacitance	Current	Pulse Interval
1	9.143	17.915	17.073
2	18.868	18.772	22.509
3	28.816	20.140	17.246
Delta	19.672	2.225	5.436
Rank	1	3	2

**Table 10:** Mean response table for the surface microcrack density of NMTMCs.

Level	Capacitance	Current	Pulse Interval
1	0.09352	0.08349	0.12128
2	0.12244	0.08328	0.08159
3	0.06365	0.11284	0.07674
Delta	0.05879	0.02956	0.04454
Rank	1	3	2

**Table 11:** Mean response table for the surface microcrack density of Ti6Al4V.

Level	Capacitance	Current	Pulse Interval
1	0.12192	0.08455	0.08170
2	0.06259	0.07114	0.08054
3	0.04349	0.07231	0.06576
Delta	0.07843	0.01314	0.01594
Rank	1	3	2

**Table 12:** Single-factor experiment on discharge capacitance.

Factor/Level	Discharge Capacitance (kpF)	Charging Current (A)	Pulse Interval (μs)
1	10	0.11	40
2	50	0.11	40
3	100	0.11	40
4	150	0.11	40
5	200	0.11	40

**Table 13:** Single-factor experiment on charging current.

Factor/Level	Charging Current (A)	Discharge Capacitance (kpF)	Pulse Interval (μs)
1	0.11	10	40
2	1.55	10	40
3	3.11	10	40
4	4.11	10	40
5	5.25	10	40

**Table 14:** Single-factor experiment on pulse interval.

Factor/Level	Pulse Interval (μs)	Discharge Capacitance (kpF)	Charging Current (A)
1	1	10	0.11
2	10	10	0.11
3	20	10	0.11
4	30	10	0.11
5	40	10	0.11

## Data Availability

Data are contained within the article.
